# A Urine-Based Liquid Biopsy Method for Detection of Upper Tract Urinary Carcinoma

**DOI:** 10.3389/fonc.2020.597486

**Published:** 2021-02-09

**Authors:** Yansheng Xu, Xin Ma, Xing Ai, Jiangping Gao, Yiming Liang, Qin Zhang, Tonghui Ma, Kaisheng Mao, Qiaosong Zheng, Sizhen Wang, Yuchen Jiao, Xu Zhang, Hongzhao Li

**Affiliations:** ^1^ Department of Urology, The First Medical Center of Chinese PLA General Hospital, Beijing, China; ^2^ Department of Urology, The Sixth Medical Center of Chinese PLA General Hospital, Beijing, China; ^3^ Department of Urology, The Seventh Medical Center of Chinese PLA General Hospital, Beijing, China; ^4^ Department of Urology, The Fourth Medical Center of Chinese PLA General Hospital, Beijing, China; ^5^ Genetron Health (Beijing) Technology, Co. Ltd., Beijing, China; ^6^ State Key Lab of Molecular Oncology, National Cancer Center/National Clinical Research Center for Cancer/Cancer Hospital, Chinese Academy of Medical Sciences and Peking Union Medical College, Beijing, China

**Keywords:** hematuria, liquid biopsy, next-generation sequencing, methylation, upper tract urinary carcinoma, logistic regression model

## Abstract

**Background:**

Conventional clinical detection methods such as CT, urine cytology, and ureteroscopy display low sensitivity and/or are invasive in the diagnosis of upper tract urinary carcinoma (UTUC), a factor precluding their use. Previous studies on urine biopsy have not shown satisfactory sensitivity and specificity in the application of both gene mutation or gene methylation panels. Therefore, these unfavorable factors call for an urgent need for a sensitive and non-invasive method for the diagnosis of UTUC.

**Methods:**

In this study, a total of 161 hematuria patients were enrolled with (n = 69) or without (n = 92) UTUC. High-throughput sequencing of 17 genes and methylation analysis for *ONECUT2* CpG sites were combined as a liquid biopsy test panel. Further, a logistic regression prediction model that contained several significant features was used to evaluate the risk of UTUC in these patients.

**Results:**

In total, 86 UTUC− and 64 UTUC+ case samples were enrolled for the analysis. A logistic regression analysis of significant features including age, the mutation status of *TERT* promoter, and *ONECUT2* methylation level resulted in an optimal model with a sensitivity of 94.0%, a specificity of 93.1%, the positive predictive value of 92.2% and a negative predictive value of 94.7%. Notably, the area under the curve (AUC) was 0.957 in the training dataset while internal validation produced an AUC of 0.962. It is worth noting that during follow-up, a patient diagnosed with ureteral inflammation at the time of diagnosis exhibiting both positive mutation and methylation test results was diagnosed with ureteral carcinoma 17 months after his enrollment.

**Conclusion:**

This work utilized the epigenetic biomarker *ONECUT2* for the first time in the detection of UTUC and discovered its superior performance. To improve its sensitivity, we combined the biomarker with high-throughput sequencing of 17 genes test. It was found that the selected logistic regression model diagnosed with ureteral cancer can evaluate upper tract urinary carcinoma risk of patients with hematuria and outperform other existing panels in providing clinical recommendations for the diagnosis of UTUC. Moreover, its high negative predictive value is conducive to rule to exclude patients without UTUC.

## Introduction

Upper tract urinary carcinoma (UTUC) including renal pelvic cancer and ureteral cancer accounts for approximately 5% of urothelial carcinomas (1, 2). During its diagnosis, patients are subjected to extensive examinations, including endoscopy, imaging of the urinary tract, and cytology or FISH testing. However, a few cases still cannot be accurately diagnosed. Before surgery, ureteroscopy is the only standard method applied to acquire the pathological status of the samples (3, 4). This method, nevertheless, is an invasive procedure that can only be performed in the hospital by experienced doctors. Besides causing discomfort and pain, it causes complications such as severe infections, i.e., 4%~25% as documented and even prophylactic use of antibiotics (5). Besides, the risk of implantation and dissemination of tumor cells might be encountered during the procedure (6). As diagnostic tools, cytology, and FISH are non-invasive yet display low sensitivity (7). Generally, an effective diagnostic method is imperative for the appropriate treatment of UTUC.

With the advent of next-generation sequencing in the last decade, biomarker searching became much easier, and multiple driver gene variations have been identified in urinary carcinoma. Of note, high rates of activating mutations in the upstream promoter of the *TERT* gene were found in the majority of upper tract urinary carcinomas and bladder cancers (BCs) (8–10). Additionally, important oncogene mutations by *FGFR3*, *HRAS*, *KRAS*, and *PIK3CA* occur at high frequency in non-muscle-invasive BCs (11–13). While mutations by *TP53*, *CDKN2A*, *MLL*, and *ERBB2* genes are frequently found in muscle-invasive BCs and UTUC (14–16). Unlike in UTUC research, molecular diagnostic methods have performed effectively in BC research. For instance, the diagnostic sensitivity of the UroSEEK method detecting mutations by 11 genes in UTUC was only 75%, much lower than 95% in BC (17).

DNA methylation, which is associated with the loss of gene expression occurs prevalently in patients diagnosed with urothelial cancer. In a previous study conducted in China, the methylation status of 10 selected genes among them, *ABCC6*, *BRCA1*, *CDH1*, *GDF15*, *HSPA2*, *RASSF1A*, *SALL3*, *THBS1*, *TMEFF2*, and *VIM* was tested during the detection of BC and UTUC. Results suggested a sensitivity and specificity of 0.82 and 0.68, respectively, among the UTUC cohort, which was insufficient for clinical application (18).

Based on the findings reported above, a more reliable biomarker is needed to advance the diagnosis of UTUC. Herein, we evaluated the performance of the *ONECUT2* methylation test in the detection of UTUC. To further improve the sensitivity of this tool, commonly occurring mutations of 17 genes in urothelial cancer were added into our test panel.

## Materials and Methods

### Patients and Samples

This double-blind and prospective clinical trial was started in 2017. Between October 2017 and May 2018, all urine samples were collected from patients without a history of any malignant disease in recent 5 years and with microscopic or macroscopic hematuria from three hospitals (The First, the Fourth and The Seventh Medical Center of Chinese PLA Navy General Hospital). Informed written consent was obtained from patients at PLA General Hospital and the study was approved by the Committee on Clinical Research Ethics of the Chinese PLA General Hospital. A total of 69 hematuria patients diagnosed with malignant UTUC (UTUC+) while the other 92 hematuria patients that were diagnosed with non-malignant UTUC (UTUC−) were enrolled respectively. All the enrolled patients were examined by endoscopy, abdomen ultrasound, CT scan, and MRI of abdomen and pelvis. Using these clinical standard diagnostic methods, no malignant tumor was found in UTUC− patients. At the same time, considering the slight limitation of the sensitivity of these methods, we followed up the UTUC− group for about 2 years to exclude undetected tumors. Correspondingly, all 69 malignant patients’ diagnosis results had been confirmed by histopathological methods after surgical treatments.

In total, 50-ml first-void Urine sample was processed within 12 h after collection. The samples were centrifuged at 2000g for 10 min, then the pellet was once washed with phosphate-buffered saline, and stored at −80°C until DNA extraction. Twelve tissue samples were effectively collected for validation, immersed in an RNA later solution (Thermo Fisher, Cat. No. AM7022) and stored following the manufacturer’s instruction until DNA extraction. The tests were blinded to the clinical data of the patients.

### Next-Generation Sequencing Analysis

#### Amplicon-Based Sequencing Design

The panel of Genetron-health 17 genes (**Supplementary File 1**) was designed to maximize the number of unique driver gene variants of UTUC by a limited number of amplicons. The regions were selected in reference to the results of previous research (17, 19, 20). In total, 38 pairs of primers were selected using a customized procedure to balance coverage, Tm, dimmer potential, and predicted specificity with the human genome (Cancer Gene Considerable Cover algorithm).

#### Multiplex PCR-Based Next-Generation Sequencing

Primers for several segments of the 17 genes in the first and second enrichments were designed separately. They were synthesized by Sangon Biotech and dissolved to 100 μmol/L with low TE buffer. Sequencing libraries were generated using multiplex PCR methods (primers and reaction conditions are described in **Supplementary File 1**). Subsequently, 20ul pooled amplicons were sequenced on the Ion Proton system (Thermo Fisher Scientific).

#### Data Analysis and Workflow

Local alignments of reads to the hg19 genome were performed using bowtie2 (version 2.2.4) in paired-end mode. SAM alignment files were converted to BAM files, sorted and indexed using Samtools (version 0.1.19). BAM files were processed with bam-read count and the outputs were processed with a custom-written Perl script. Normal SNP variant mutation frequency is usually at around 50%, here, the frequency was set at >0.5% and supporting unduplicated reads at ≧20 as an abnormal cutoff to distinguish the variants appeared in the detection.

### Methylation Analysis

This assay was designed to detect CpG-sites on the *ONECUT2* gene, it was performed using EZ DNA Methylation-Lightning™ Kit (Zymo Research Corporation, Irvine, California, USA), according to the manufacturer’s protocol. Briefly, bisulfite-specific real-time PCR was designed for 20-ng bisulfite transformed DNA. Ct values representing the relative quantity of methylated and unmethylated parts were separately measured by FAM and VIC signals, and the delta ct values were calculated as methylation score.

### Statistical Analysis and Logistic Regression Model

Statistical analysis was performed using Python (Version 3.6) with scipy (1.1.0) and scikit-learn (0.19.2) module. P-values at P < 0.05 were considered statistically significant. Univariate logistic regression analysis was used to calculate the association between UTUC and the diagnostic variables.

A logistic regression model was constructed from the training cohort of 115 samples by random sampling. The model performance was evaluated both on the training and validation data sets, by the area under the curve (AUC) statistics. The sensitivity and specificity of the model were also determined using an optimized cutoff value which was applied using Youden’s index. Cross-validated coefficients for each feature using logistic regression have been given. The model was initiated in R package ‘glmnet’ (R version 3.5.1), and the penalty parameter alpha was optimized with 10-fold cross-validation within the training data set and the optimized value was 0.

## Results

### Patient Demographics

**Figure 1:**
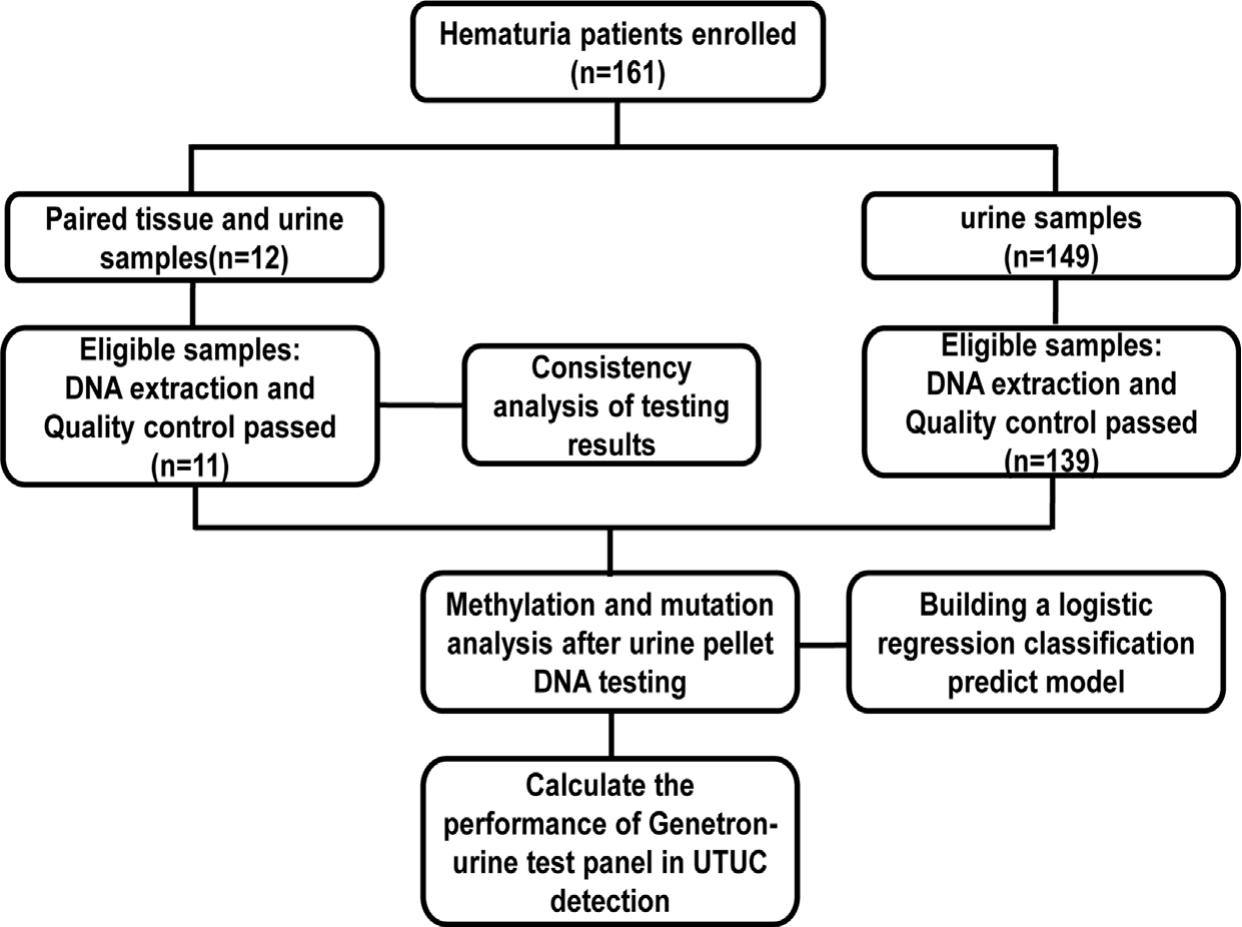
Sample and data processing work-flow.

In total, 150 (93.2%) of the urine samples and 11 (91.7%) of tissue samples passed the quality control for further testing (**Figure 1**). Overall, 107 males and 43 females were enrolled as subjects, with a median age of 60 (range from 18 to 88) years. Patients and tumor characteristics are described in **Table 1**. In 64 cases, UTUC was confirmed as a cause of hematuria while the cause of the remaining 86 patients was found to be non-malignant. Patients diagnosed with UTUC were significantly older compared to non-malignant patients (p < 0.01, **Table 2**). FISH tests were only performed on 80% (n = 51) of UTUC+ and 9% (n = 8) of UTUC− patients. The sensitivity and specificity of FISH were 51% (26/51) and 100% (0/8) respectively.

**Table 1:** Clinical and histopathological characteristics of enrolled cases.

Characteristic	Number of UTUC+ patients(N = 64)	Number of UTUC− patients(N = 86)
Age, y		
Median (range)	67(26~88)	56(18~82)
Gender, n (%)		
Male	40(62.5)	67(77.9)
Female	24(37.5)	19(22.1)
FISH, n (%)		
Positive	26(40.6)	0(0.0)
Negative	25(39.1)	8(9.3)
Grade, n (%)		
Low grade High grade	17(26.6) 47(73.4)	- -
Type, n (%)		
NMIUC	22(34.4)	–
MIUC	39(60.9)	–

FISH, Fluorescence in situ hybridization; NMIUC, Non-muscle-invasive urothelial carcinoma; MIUC, Muscle-invasive urothelial carcinoma.

**Table 2:** Univariable logistic regression analysis including significant features.

Variables	OR*	95% CI	P value
Gender			
male	0.47	0.23~0.97	0.04(<0.05)
Age, y	1.08	1.04~1.12	<0.01
>50			
Mutations			
*FGFR3*	28.33	3.64~220.31	<0.01
*TERT*	37.06	8.39~163.74	<0.01
*PIK3CA*	7.2	0.82~63.25	0.075
*TP53*	8.47	2.33~30.73	<0.01
Methylation			
*ONECUT2*(△ct<7.93)	131.91	39.87~436.49	<0.01
Panel			
≥1 gene mutated or the *ONECUT2*gene methylated	133.17	40.26~440.45	<0.01

*OR was defined as UTUC risk of each feature.

### The Concordance Profiling Between Urine ctDNA and Matched Tumor Tissues

**Figure 2:**
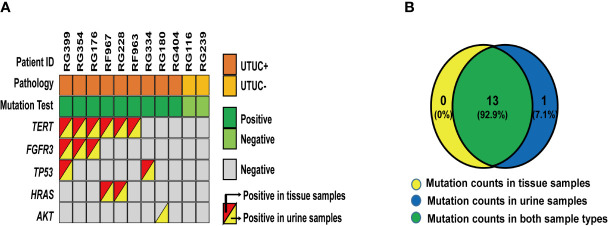
Variants detected in 12 paired urine and tissue samples: **(A)** A heatmap shows variants detected in 12 paired urine and tissue samples. **(B)** A Venn diagram shows the relationship of these 26 variants from each set of different types of samples.

The consistency of mutations in urine samples and the corresponding tissue samples were evaluated to confirm the sources of these variants. As a result, a total of 12 matched tissue samples were effectively collected and 11 qualified DNA samples were identified. As shown in **Figure 2**, 14 variants from 5 genes were detected from 9 UTUC+ samples where 13 of them were positive in both types of samples. In the case of RG180, *AKT* mutation was shown from urine other than tissue samples indicating the effectiveness of urine samples as a supplement for genetic analysis of UTUC tissue samples in instances where genetic heterogeneity is considered as an issue. In addition, no mutations were detected in the urine and tissue samples of the two UTUC− patients. In summary, the concordance rate of variant detection between urine and tissue samples was 93% (13/14).

### Univariate Logistic Regression of Significant Features

Univariate analysis was performed for each of these variants as well as clinical factors to assess the strength of these factors in evaluating UTUC risk by calculating the odds ratios (**Table 2**). Mutated or methylated Gene including *FGFR3*, *TERT*, *TP53*, *ONECUT2*, and age older than 50 showed a significant impact in evaluating UTUC risks (P-value < 0.01). And the superiority of the panel was witnessed in the integration of all these markers (≥1 of 17 genes mutated or *ONECUT2* CpG methylated).

### Gene Mutations Results of Urine Samples Were Consistent With Characteristics of Previous UTUC Mutation and Provided New Clinical Potential Applications

**Figure 3:**
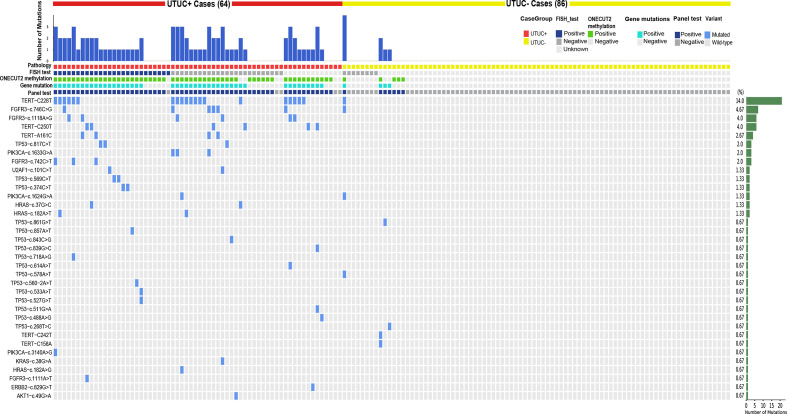
Heatmap of all variations in urine samples: it demonstrates the variants detected in each case’s urine sample.

To better understand how each variant contributes to this panel, a heatmap was drawn in **Figure 3**. Despite this panel covering hot-spots mutations of 17 genes, only variants from nine genes showed positive mutations. *TERT* C228T, *FGFR3* c.746C>G, c.1118A>G, and *TERT* C250T were on the top 4 of the list with a long tail of several other mutations from *ERBB2*, *HRAS*, *KRAS*, *PIK3CA*, *TP53*, *U2AF1*, and *AKT1* (**Supplementary Table 1**). This distribution pattern of driver genes corroborates with previous research in the sense that *TERT*, *FGFR3*, *TP53*, *PIK3CA*, and *RAS* genes exhibited high frequencies in the UTUC mutation landscape. FISH test results were shown along with the panel, notably, the sensitivity of the FISH test was low [about 51% (26/51)], but with perfect specificity and no false-positive found from the 8 tested UTUC− samples (**Table 1**). With the mutation test, the sensitivity and specificity were 71.9% and 91.4% respectively (**Table 3**), which was close to the sensitivity of 75% in the UTUC diagnostic cohort in a previous study that solely used mutant genes panel (19). Therefore, these data confirm the limitation of sensitivity when gene mutation detection was used solely in the diagnosis of UTUC.

**Table 3:** Comparison of detection performance when using gene mutations solely, *ONECUT2* methylation solely and panel test.

	Test performance			
	Sensitivity(95%CI)	Specificity(95%CI)	PPV(95%CI)	NPV(95%CI)
*ONECUT2 methylation	89.1%(79.1%~94.6%)	94.2%(87.1%~97.5%)	91.9%(82.5%~96.5%)	92.0%(84.5%~96.1%)
**Gene Mutations	71.9%(59.9%~81.4%)	95.4%(88.6%~98.2%)	92.0%(81.2%~96.9%)	82.0%(73.3%~83.3%)
***Panel	92.2% (83.0%~96.6%)	91.9%(84.1%~96.0%)	89.4%(79.7%~94.8%)	94.1% (86.8%~97.4%)

*ONECUT2 methylation positive: △Ct value is smaller than the cutoff value(7.93).

**Mutation positive: ≥1 of 17 genes are mutated.

***Panel positive: At least one of ONECUT2 methylation test and mutation test is positive.

**Figure 4:**
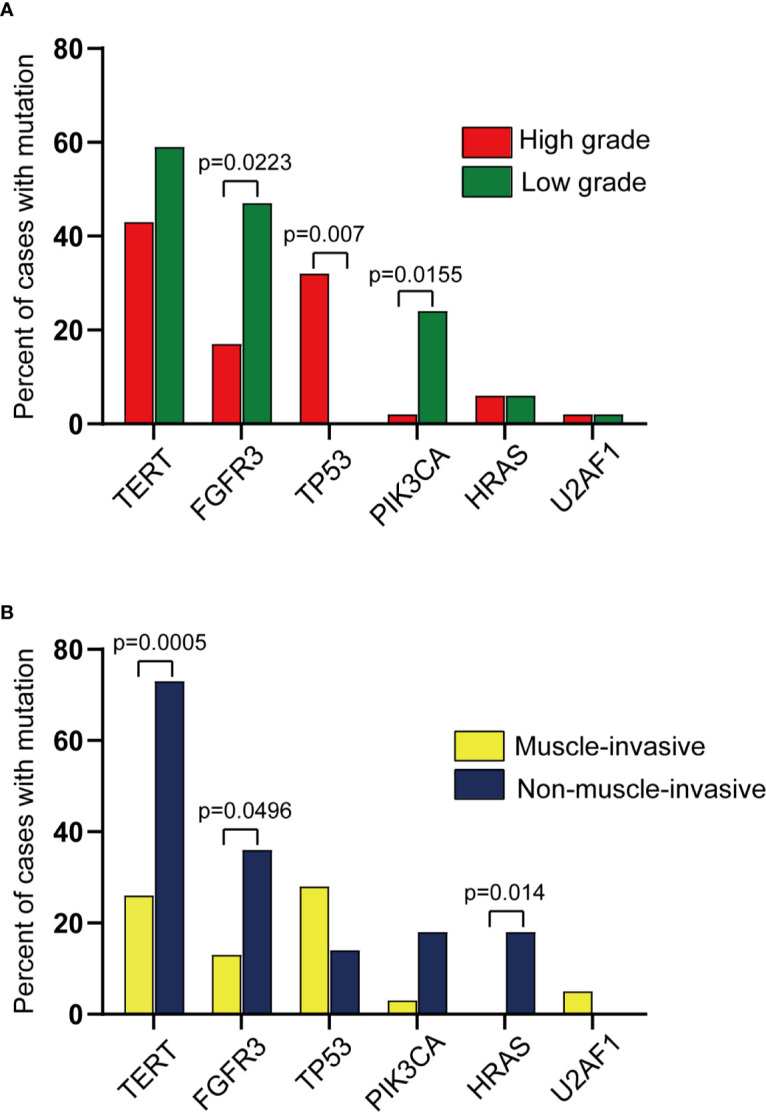
Comparison of mutations across different groups profiled in this study. (Pairwise comparison results from Fisher’s exact test). **(A)** Comparison of mutations across high vs. low grade UTUC. **(B)** Comparison of mutations across muscle-invasive vs. non-muscle-invasive UTUC.

When the mutation detection results were analyzed solely, there was a significant difference between the muscle-invasive group and the non-muscle-invasive group (**Supplementary Table 2**, p value = 0.037). Additionally, a significantly higher frequency of *TP53* mutations in high versus low-grade samples (31.9% vs. 0%; p = 0.0065, Fisher’s Exact Test) was observed, and conversely, found disproportionately more *FGFR3* mutations (47.1% vs. 17.0%; p = 0.0223, Fisher’s Exact Test) and *PIK3CA* mutations (23.5% vs. 2.1%; p = 0.0155, Fisher’s Exact Test) in low versus high-grade cases (**Figure 4A**). Likewise, a significantly higher frequency of *TERT* promoter mutations (72.7% vs. 25.6%; p = 0.0005, Fisher’s Exact Test) and *HRAS* mutations (0% vs. 18.2%; p = 0.014, Fisher’s Exact Test) was evident in non-muscle-invasive versus muscle-invasive samples for the first time in UTUC cohort (**Figure 4B**). This thus reflected the significance of adding detection of gene mutation to our test panel, which could provide evidence for the classification of UTUC+ patients.

### *ONECUT2* Methylation Exhibited a Satisfactory Performance as a Diagnostic Biomarker of UTUC

**Figure 5:**
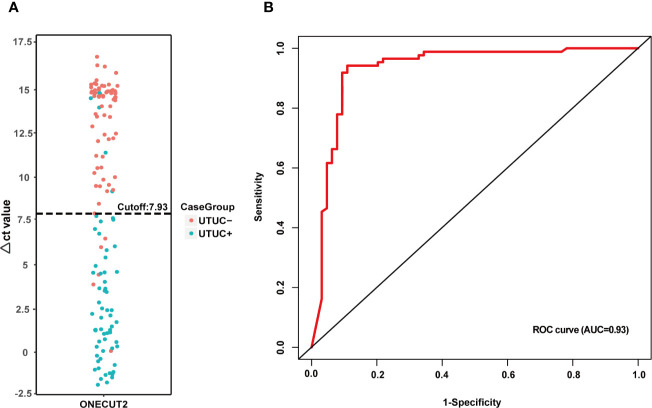
Performance analysis of *ONECUT2* methylation: **(A)** The *ONECUT2* methylation Δct-value distribution of different types of samples. **(B)** ROC curve of the *ONECUT2* methylation (AUC = 0.92) also indicating the optimized Δct value cutoff is at 7.93 in this study.

The analysis was performed to confirm the best cutoff of *ONECUT2* methylation status (**Figure 5**). The Δct value of *ONECUT2* in all urine samples is shown in **Figure 5A**. With a cutoff of 7.93, the *ONECUT2* methylation detection ability was the largest, displaying the AUC of 0.93 (**Figure 5B**). With the singly use of the *ONECUT2* methylation test in this cutoff value, genetic abnormalities in 89.1% (57/64) urine of UTUC+ patients and 5.8% (5/86) of UTUC− group were detected resulting in a sensitivity of 89.1% (57/64), and a specificity of 94.2% (81/86) (**Table 3**). This performance of the *ONECUT2* methylation test was better than the one reported previously (sensitivity of 82% and specificity of 62% with a panel of *VIM*, *RASSF1A*, *GDF15*, and *TMEFF2* methylation in UTUC group) (18).

### The Performance of the Test Panel in UTUC Detection Showed Higher Sensitivity and NPV

By combining *ONECUT2* methylation and gene mutation results as a UTUC diagnostic test panel (≥1 of 17 genes mutated or *ONECUT2* CpG methylated showed a positive result), the performance of the test improved, the sensitivity of this test panel rose to 92.2% (59/64), and the specificity was 91.9% (79/86). Simultaneously, the panel demonstrated a positive predictive value of 89.4% and a negative predictive value of 94.1% (**Table 3**). Moreover, by combining the detection results of gene mutations with *ONECUT2* methylation, the sensitivity was further improved. It was worth noting that the double-positive result (≥1 of 17 genes mutated and *ONECUT2* CpG methylated) potentially reveal a higher risk of UTUC. By the time the article was being written, almost all patients enrolled had completed a two-year follow-up, and two patients named RH645 and RG342 (**Supplementary Table 1**) with double-positive test results in UTUC− cohort were focused. It was found that patient RG342 was diagnosed with ureteral cancer in May 2019. Notably, a close follow-up of patient RH645 was still ongoing.

### Comparison of Multivariate Logistic Regression Models Prompted the Direction of Panel Optimization

**Figure 6:**
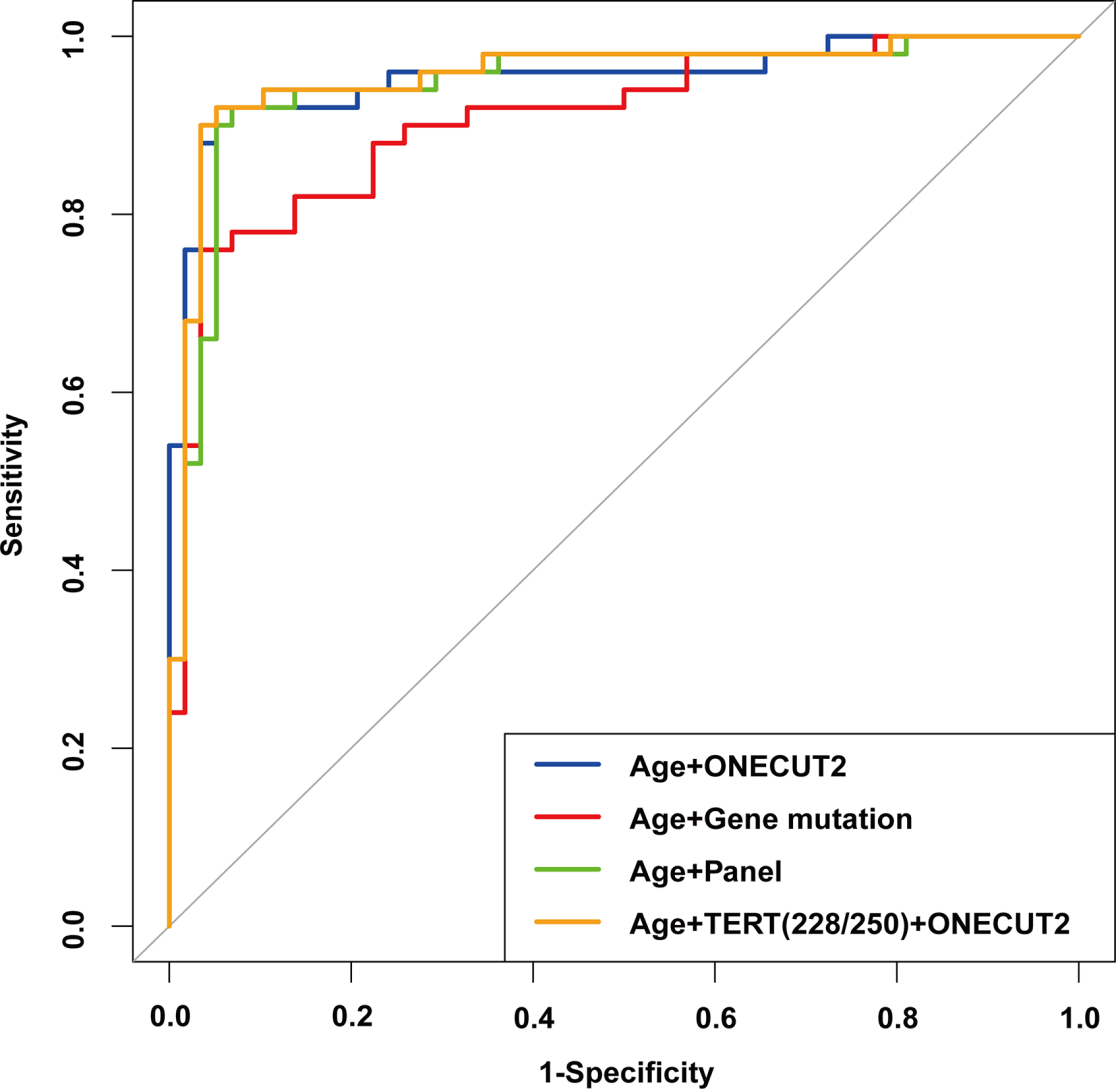
ROC of the multivariable logistic regression models with different features in the training set.

Out of the 150 samples, 108 were randomly selected as the training set, and the remaining 42 samples were the validation set. Based on the results of Univariate logistic regression, significant features were combined to construct 4 logistic regression models. From the ROC curve shown in **Figure 6**, the model constructed with the features of age and the mutation status of *TERT* promoter (mutation of at least one hotspot on *TERT* g.1295228C>T and g.1295250C>T) and *ONECUT2* methylation level (Model D) had the largest AUC of 0.957, whereas the AUC of other three models were 0.947 (age and panel test results, Model C), 0.953 (age and *ONECUT2* methylation, Model A), and 0.903 (age and 17 genes mutation test result, Model B). By selecting the optimal cutoff according to the highest Youden index in each of the four models, the model with age, mutation status of *TERT* promoter and *ONECUT2* methylation level showed an optimal performance with a sensitivity of 94.0%, a specificity of 93.1%, a PPV of 92.2% and an NPV of 94.7% (**Table 4**). This model maximized sensitivity without a major reduction in specificity hence was considered optimal. And in the validation set, a prediction using the above features were completed and obtained an AUC of 0.962 (**Supplementary Figure 1**).

**Table 4:** Effect on sensitivity, specificity, PPV and NPV at the respective cutoff of different models.

Variables of models	Models with different features			
	Model A	Model B	Model C	Model D
AUC of training set	0.953(0.911~0.996)	0.903(0.844~0.962)	0.947(0.901~0.992)	0.957(0.916~0.999)
Cutoff	0.408	0.497	0.498	0.412
Sensitivity(%)	92.0(81.2~96.9)	76.0(62.6~85.7)	92.0(81.2~96.9)	94.0(83.8~97.9)
Specificity(%)	94.8(85.9~98.2)	96.6(88.3~99.1)	93.1(83.6~97.3)	93.1(83.6~97.3)
PPV (%)	93.9(83.5~97.9)	95.0(84.5~98.6)	92.0(81.2~96.9)	92.2(81.5~96.9)
NPV (%)	93.2(83.8~97.3)	82.4(71.6~89.6)	93.1(83.6~97.3)	94.7(85.6~98.2)

Features in different models as follows:

Model A: age+ Onecut2 methylation.

Model B: age+ gene mutations.

Model C: age+ panel test.

Model D: age+ TERT C228T/C250T+ Onecut2 methylation.

PPV, Positive predictive value; NPV, Negative predictive value.

95% CI values were showed in brackets.

## Discussion

The accurate distinction between benign and malignant tumors in the diagnosis of UTUC from a large number of hematuria patients remains a clinically challenging issue. Herein, we evaluated the performance of *ONECUT2* methylation detection in the UTUC diagnostic cohort for the first time, with mutations of 17 genes being combined into the test panel. Surprisingly, this is the first time that the superiority of this epigenetic biomarker *ONECUT2* has been demonstrated in UTUC diagnostic studies, and reported a high potential for clinical application compared to other methylation related methods. A logistic regression prediction model based on liquid biopsy of gene variants and clinical factors was screened for the accurate diagnosis of UTUC patients presenting microscopic or macroscopic hematuria. This will enable urologists to adjust the examination or treatment plan of the patient according to the risk, thereby reducing the discomfort and minimizing the cost incurred by the patient. In the current study, the test panel demonstrates high sensitivity and NPV for detecting patients with a high risk of UTUC as well as accurately excluding patients with benign hematuria. Specifically, the performance of our test panel shows a comprehensive improvement compared to previous studies which were based on either gene mutation panel or genes methylation panel.

So far, numerous studies have focused on the use of molecular tests in the diagnosis of urinary carcinoma in patients presenting hematuria, and a handful of these assays have already been approved by FDA (21–25). However, the effectiveness of cytology and imunocytology highly depends on the skills and experience of the pathologist and not the efficacy of the method (26). Generally, the FISH tests from previous research have usually been reporting a sensitivity of about 70%~80% in detecting urothelial cell carcinoma (8), nevertheless, the FISH test in our study was unable to obtain sensitivity in nearly half of UTUC+ cases (25/51). This explains its limitation in clinical practice, however, for a reliable conclusion, more experiments with larger sample sizes are necessary. On the other hand, we noted a single case of FISH positive UTUC+ with a negative panel test (RH350), this test result implies that our panel design can be improved by adding more content of chromosome structure variation or gene copy number variation detection.

After further analysis, an inference emerged that multiple gene mutations detected in a few patients with false-negative test results potentially indicate the need for close follow-up. After a lower ureterectomy, a patient with hematuria in our cohort was clinically diagnosed with ureteral inflammation in December 2017 through pathology testing. Furthermore, it was found that his *TERT*, *FGFR3*, *TP53*, and *PIK3CA* genes were mutated respectively and the *ONECUT2* methylation result was positive. As a consequence, regular follow-up on this patient was performed and diagnosed him with ureteral carcinoma in May 2019, suggesting that the changes in urine genomics potentially precede the changes in imaging and can detect minimal tumor existence beyond the surgical site. Therefore, patients with a double-positive result in testing needed regular follow-ups.

In tumor grade analysis, the proportion of patients with *TERT* promoter mutations was higher in low-grade UTUC than that in high-grade ones, which was in agreement with the results of the previous UTUC cohort studies (17). And *TERT* promoter mutations status showed a significant difference in muscle-invasive and non-muscle-invasive UTUC samples. However, this is a newly discovered conclusion that needs to be validated by large-scale research. Meanwhile, genes such as *TP53* and *FGFR3* also showed their roles in predicting the grade of UTUC, which further reflected the value of our gene mutation testing. Also, it is worth noting that a non-invasive urine biopsy test has shown its potential ability in predicting tumor grade and the risk of UTUC.

*TERT* promoter mutations that had been previously reported to be closely related to UTUC were indeed significant factors in our cohort. For instance, an optimized model that only combined *TERT* promoter mutation with *ONECUT2* methylation and age yielded a satisfactory performance in the prediction of the samples. This suggests that reducing the genes to be tested in the optimized product significantly reduces the cost and time for testing as well as maintaining high accuracy. Nevertheless, this optimization of testing panel calls for validation in a larger cohort.

Again, since the relationship between age and cancer has been observed, cutoffs based on age stratification should be considered in further studies. Besides, the majority of BC share similar histogenesis as UTUC, therefore we propose that this panel test should be externally validated in a larger prospective patient cohort which includes more patients with benign and malignant bladder disease.

Furthermore, this cohort excluded information on follow-ups in a few of the enrolled patients, particularly the ones diagnosed benignly in their first testing. This attempts to answer these questions: (1) can this panel be also utilized in clinical follow-up visits to minimize the times of unnecessary invasive operations for postoperative patients or reveal the recurrence in a much more convenient way; and (2) which of these frequently mutated genes or variants can be biomarkers for prognosis prediction or even indicators of different treatment choices.

## Conclusion

In conclusion, we utilized the epigenetic biomarker *ONECUT2* for the first time in the detection of UTUC and discovered its superior performance. As a result, we developed an accurate testing panel combined with mutation of significant genes. Results suggested that this panel might result in a less extensive examination of low-risk patients and due to its high NPV, it reduces costs and discomfort among patients. Therefore, this panel provides clinicians with important predictions in addition to imaging and routine urine cytology analysis to significantly advance the diagnostic precision of UTUC. Meanwhile, a more precise disease management plan should be set up upon the discovery of a high-risk UTUC. Further validation in a large prospective cohort of a broad population is vital to confirm the true clinical value of this newly developed method.

## Data Availability Statement

The original contributions presented in the study are publicly available. This data can be found here: https://www.biosino.org/node/, accession number OEP001778.

## Ethics Statement

The studies involving human participants were reviewed and approved by Medical Ethics Committee of PLA General Hospital. The patients provided their written informed consent to participate in this study. Written informed consent was obtained from the individuals for the publication of any potentially identifiable images or data included in this article.

## Author Contributions

HL and XZ designed and supervised the study. YX and YL searched the literatures. YX, XM, XA, JG, YL, TM, SW, and YJ participated in data acquisition. YX, XA, JG, YL, and KM did the data analysis and interpretation. YX, YL, TM, and JG drafted the manuscript. YX, YL, and YJ did statistical analysis. YX and YL gave critical revision of the manuscript for important intellectual content. All authors contributed to the article and approved the submitted version.

## Conflict of Interest

YL, QZ, TM, KM, QZ, and SW were employed by the company Genetron Health (Beijing) Technology, Co. Ltd.

The remaining authors declare that the research was conducted in the absence of any commercial or financial relationships that could be construed as a potential conflict of interest.

## Supplementary Material

Supplementary Figure 1 ROC of the optimal model with the features of age and panel test results in the validation set.
