# Supplementary material for: A Urine-Based Liquid Biopsy Method for Detection of Upper Tract Urinary Carcinoma
Source: Front Oncol. 2021 Feb 9;10:597486. doi: 10.3389/fonc.2020.597486 (PMC7901537; doi:10.3389/fonc.2020.597486)
Supplement: Supplementary file 2 [file Table_2.docx]

| group | Group counts | | OR | 95% CI | P value |
| --- | --- | --- | --- | --- | --- |
|  | Mutation+ | Mutation- |  |  |  |
| High grade | 34 | 13 | 0.805 | 0.222~2.915 | 0.506 |
| Low grade | 13 | 4 |  |  |  |
|  |  |  |  |  |  |
| Muscle-invasive | 24 | 15 | 0.253 | 0.064~1.002 | 0.037(＜0.05） |
| Non-muscle-invasive | 19 | 3 |  |  |  |

**Supplementary Table 2 Fisher’s Exact Test between different clinical groups (grade or stage) using gene mutations results**

Mutation+: ≥1 gene mutated;

Mutation-: no gene mutated.
