# Supplementary material for: A Urine-Based Liquid Biopsy Method for Detection of Upper Tract Urinary Carcinoma
Source: Front Oncol. 2021 Feb 9;10:597486. doi: 10.3389/fonc.2020.597486 (PMC7901537; doi:10.3389/fonc.2020.597486)
Supplement: Supplementary file 3 [file Table_3.docx]

17 Gene included in the Panel:

*AKT1, ASXL2, CREBBP, ERBB2, ERBB3, ERCC2, FBXW7, FGFR3, HRAS, KDM6A, KRAS, PIK3CA, RHOA, SF3B1, TP53, TERT* and *U2AF1*

Primer:

| Gene | Forward Primer | Reverse Primer |
| --- | --- | --- |
| AKT1 | gggtctgacgggtagagtgt | ccgctccttgtagccaatga |
| ASXL2 | gtaagctttccttggcttattctttgac | agagcacttaccttctgagagtct |
| CREBBP | agttaaagaggaagaagagagtagcagtaa | catggaatcctaacaccgtggaa |
| ERBB2 | attgttcagcgggtctccatt | ctacgtgctcatcgctcaca |
| ERBB2 | ctcacagggacaggcagtcaca | ccacttcaaccacagtggcat |
| ERBB2 | tgctgtcacctcttggttgtg | acggtaatgctgctcatggt |
| ERBB2 | gccgtcgcttgatgaggatc | cccaaactagccctcaatccc |
| ERBB2 | catcagaactgccgaccaca | ggaaggtgaaggtgcttggat |
| ERBB2 | agccatagggcataagctgtg | ctcccataccctctcagcgta |
| ERBB3 | agttcacaaatccatcaatgttgctc | tgtaaagagacagcctttcctctga |
| ERBB3 | gcccatttttatctacttccatcttgtca | ggttccattggtagctggtgat |
| ERCC2 | aaggccgtcgtggtcttcg | gcagacgttgtctggagaagg |
| FBXW7 | tggagcatatgattttatggtaaaggtgt | gattcatcaggagagcatttaaggga |
| FBXW7 | gaatgcagagactggagaatgtataca | ggcccaaattcaccaataatagagg |
| FGFR3 | ggaactccacgtcgctgc | gcccctgagcgtcatctg |
| FGFR3 | gcaggcagctcgagct | ccaacctgcccctgct |
| FGFR3 | accaccaggatgaacaggaag | ccaggccaggcctcaa |
| HRAS | ttggacatcctggataccgcc | ctgtactggtggatgtcctcaaaa |
| HRAS | aggagcgatgacggaatataagc | cgccaggctcacctctatag |
| KDM6A | cccagtaaaaacatttatgtgaaaagccaa | cgcactcactctacctcataacc |
| KRAS | tattataaggcctgctgaaaatgactgaat | tacctctattgttggatcatattcgtcca |
| PIK3CA | accaatttctcgattgaggatcttttct | gtgttactcaagaagcagaaaggga |
| PIK3CA | cataagagagaaggtttgactgccataaa | tttgaatctttggccagtacctca |
| PIK3CA | cacttacctgtgactccatagaaaatct | ttttacagagtaacagactagctagagacaa |
| PIK3CA | gtctcaaacacaaactagagtcacaca | tcgtgcatgtgggatgtatttga |
| PIK3CA | gtggaagatccaatccatttttgttgtc | aatcttttgatgacattgcatacattcgaa |
| RHOA | gcccacagtgtttgagaactatgt | gaagaggcaaaaagctctaattctctaca |
| SF3B1 | actgattgatggtattctttatgctttcca | tgcattctagaaaaatttgcttgacaact |
| TERT | gaccccgccccgtc | gcttcccacgtgcgca |
| TP53 | cttgaaccatcttttaactcaggtactgt | gcctgggcatccttgagttc |
| TP53 | ctactgggacggaacagctt | cttgcttacctcgcttagtgct |
| TP53 | tcttgggcctgtgttatctccta | cctgacctggagtcttccagt |
| TP53 | ggcctctgattcctcactgattg | tcatagggcaccaccacactat |
| TP53 | agctgtgggttgattccaca | cagctgctcaccatcgctat |
| TP53 | gaagctcccagaatgccaga | gtagctgccctggtaggttt |
| TP53 | gttggaagtgtctcatgctggat | ccataggtctgaaaatgtttcctgact |
| TP53 | gcattctgggacagccaag | tacggccaggcattgaagt |
| U2AF1 | cccagcaaaataatcagctctcattttc | aaaaaggcaaacaaacctggcta |

Reaction:

|  |  | (ul) | Condition |
| --- | --- | --- | --- |
| Round1 | ddH2O | up to desired volume | 95℃ 2 minutes  10 cycles:  {95℃ 30s  60℃ 90s  72℃ 90s}  72℃ 10min |
|  | KAPA2G Fast PCR (Roche KK5020) Mix | 10 |  |
|  | Primer | 0.6 |  |
|  | template | 20ng |  |
|  | total | 20 |  |
|  | | | |
| Round2 | ddH2O | up to desired volume | 95℃ 5 minutes  15 cycles:  {95℃ 30s  60℃ 90s  72℃ 90s}  72℃ 15min |
|  | KAPA2G Fast PCR (Roche KK5020) Mix | 10 |  |
|  | Primer | 1 |  |
|  | enriched DNA | 5 |  |
|  | total | 20 |  |
| Each reaction was cleaned once using Agencourt AMPure XP 60mL kit (Beckman A63881) to remove unused primers, according to the manufacturer’s specifications. The concentration of the barcoded PCR produced library was then measured by Qubit 3.0(Thermo Fisher Scientific), and diluted to 100pMol. | | | |

the first-round enrichment in a 20μl reaction mixtures containing 10μl KAPA2G Fast PCR (Roche KK5020), 0.6ul primers mixtures, 20ng sample gDNA template and distilled water up to desired volume. Initial denaturation was at 95℃ 2 minutes, followed by 10 cycles at 95℃ for 30s, 60℃ for 90s, and 72℃ for 90s, and a final elongation at 72℃ for 10min. Immediately after the reaction, 5μl enriched DNA was then combined with 1μl universal index primers, 10μl KAPA2G Fast PCR, and distilled water up to a system of 20μl, the second enrichment was carried out at 95℃ for 5 minutes, followed by 15 cycles at 95℃ for 30s, 60℃ for 90s, and 72℃ for 90s, and a final elongation at 72°C for 15min.

Each reaction was cleaned once using Agencourt AMPure XP 60mL kit (Beckman A63881) to remove unused primers, according to the manufacturer’s specifications. The concentration of the barcoded PCR produced library was then measured by Qubit 3.0(Thermo Fisher Scientific), and diluted to 100pMol.
