# Supplementary figures and images for: A Urine-Based Liquid Biopsy Method for Detection of Upper Tract Urinary Carcinoma
Source: Front Oncol. 2021 Feb 9;10:597486. doi: 10.3389/fonc.2020.597486 (PMC7901537; doi:10.3389/fonc.2020.597486)

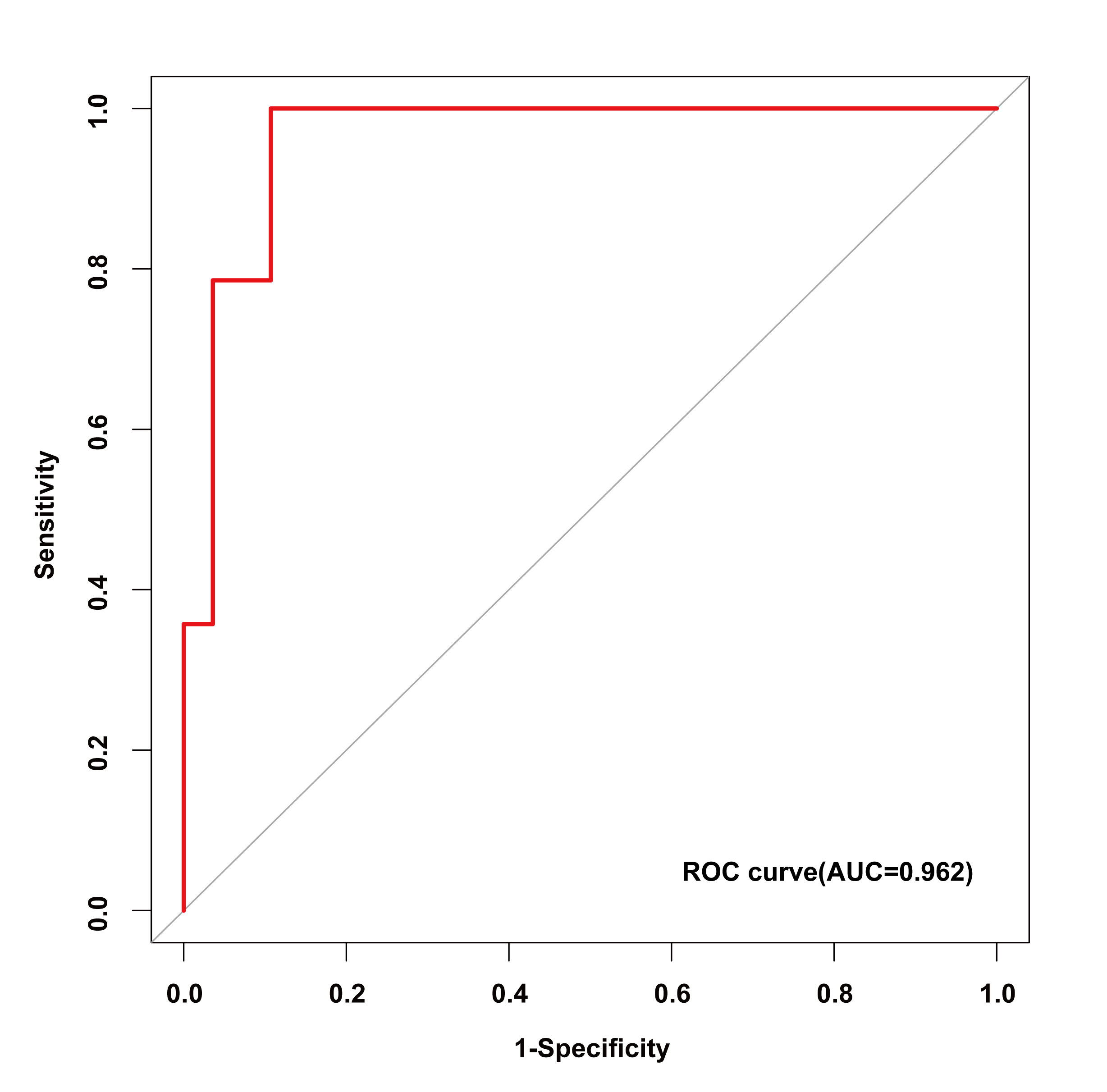

Supplement: Supplementary Figure 1 — ROC of the optimal model with the features of age and panel test results in the validation set. [file Image_1.tif]
